# Seedling-Stage Deficit Irrigation with Nitrogen Application in Three-Year Field Study Provides Guidance for Improving Maize Yield, Water and Nitrogen Use Efficiencies

**DOI:** 10.3390/plants11213007

**Published:** 2022-11-07

**Authors:** Yuxi Li, Jian Chen, Longbing Tian, Zhaoyin Shen, Daniel Buchvaldt Amby, Fulai Liu, Qiang Gao, Yin Wang

**Affiliations:** 1 College of Resources and Environmental Sciences, Jilin Agricultural University, Changchun 130118, China; 2 Key Laboratory of Straw Comprehensive Utilization and Black Soil Conservation, Ministry of Education Jilin Agricultural University, Changchun 130118, China; 3 Department of Plant and Environmental Science, Faculty of Science, University of Copenhagen, Højbakkegaard Alle 13, 2630 Copenhagen, Denmark; 4 Department of Plant and Environmental Sciences, Section for Organismal Biology, Faculty of Science, University of Copenhagen, Thorvaldsensvej 40, 1871 Frederiksberg, Denmark

**Keywords:** maize, deficit irrigation, nitrogen fertilization, soil water content, seedling growth, grain yield, water use efficiency, nitrogen use efficiency

## Abstract

Deficit irrigation (DI) was acknowledged as an effective technique to improve water use efficiency (WUE) without significant yield reduction. In this study, a 3-year field experiment was conducted in Northeast China during 2017–2019 to investigate the combined effects of 3-week DI from 3-leaf stage and N fertilization on maize seedling growth and determine the resulting impacts on silking growth and yield formation, N use efficiency (NUE) and WUE. Results showed that seedling-stage DI decreased leaf area and photosynthesis, thus significantly limited shoot and root dry biomass for maize seedling, compared to well-watered (WW) plants. In 2017 and 2019, seedling-stage DI positively improved seedling growth with higher root: shoot ratio and enhanced drought tolerance, under higher initial soil water contents (SWC) with sufficient precipitation before DI. The DI-primed plants showed similar or better performances on reproductive growth, grain yield, WUE and NUE compared to WW plants, even experiencing heavy rainfall or drought stresses around the silking stage. However, the contrasting results were observed in 2018 with negative DI effects on seedling and silking growth and final yield, probably due to less rainfall and lower SWC before DI. In all 3 years, N fertilization had significant compensatory effects on limited seedling growth under DI, and its effect was much less in 2018 than other years due to adverse early climate. The principal component and correlation analysis revealed maize silking growth, grain yield, NUE and WUE were strongly related to the seedling growth as affected by water and N managements under various climatic conditions. In conclusion, a short-term and moderate DI regime—adopted at the seedling stage under higher initial SWC and coupled with an appropriate N fertilization—is beneficial to control redundant vegetative growth while optimizing root development, therefore effectively improving drought tolerance for maize plants and achieving higher grain yield, WUE and NUE.

## 1. Introduction

Maize is one of most important crops worldwide for food, feed and industrial uses and is widely planted with about a total area of 190 million hectares, accounting for more than 38% of global cereal production in 2020 [1]. By 2050, the global maize production had estimated needs to increase by 66% to meet the enormous demand of an ever-growing population [2]. However, in recent years, maize production is negatively affected in many regions around the world due to the frequent drought stress which are associated with the increasing shortage of water resources and/or the aperiodic water deficit under uncertain and uneven precipitation [3,4]. In Northeast China (NEC), the most important maize planting area in China, maize production is facing more and more frequent drought stresses with increasing air temperature and uneven precipitation, especially at early-vegetative and reproductive growth stages [4,5]. In order to avoid drought stress and corresponding negative effects on maize growth and grain yield, more and more farmers began to adopt irrigation for maize in NEC. However, the full and excessive irrigation generally adopted by farmers largely increased water consumption under inadequate water resource condition in this region, also significantly reduced water use efficiency (WUE). In the future, the growth limitation and yield loss induced by a water deficit may be further aggravated in global maize production—as a result of the whole earth’s climate change— especially for NEC and other semi-arid and semi-humid regions [6,7]. Therefore, appropriate water management approaches are required to be adopted for the maize cropping system in these regions to effectively use agricultural water resource while maintaining or even enhancing crop productivity [8,9].

The deficit irrigation (DI) is considered as an effective water saving irrigation method and has been broadly adopted for dryland cropping regions in China and many other countries [9,10,11]. It is defined as a water management method that regulates a reduced water supply which is relative to the full water requirement for optimal crop growth during a specific growing period, to create a controlled soil water deficit condition and reduce actual evapotranspiration (ETa) while improving WUE [9,12,13]. In practice, the regulated time and degree of DI played pivotal roles in allowing DI implementation and determining its application effectiveness [10,14]. For maize, several previous researchers have found that the moderate DI application at the seedling stage leads to a little yield reduction and even could produce an equal yield level as compared to well-watered condition, and consequently obtained a higher WUE. However, the DI application at later-vegetative or reproductive growth stages generally reduced grain yields, and the yield reduction increased with increasing duration or stress degree under DI regime [15,16,17,18]. A recent global scale meta-analysis also demonstrated that the implementation of DI at the early-vegetative growth stage is more beneficial to maize production, by comprehensively assessing the DI responses of maize yield, ETa and WUE across the various varieties, climates, soil textures and fertilization practices [10]. The beneficial effects of moderate DI at the seedling stage can be explained by the enhanced plant stress resistance, reflected by higher photosynthetic capacity, rapid recovery of stomatal morphology and function, optimal activation of the antioxidant system and higher root: shoot ratio [8,9,19]. Thus, maize plants primed by the seedling-stage DI are known to have better growth performances when exposed to various environmental stresses during later growth stages, and consequently leads to higher grain yield and WUE [20,21]. Until now, the effects of DI on maize growth, physiological characteristics, grain yield and WUE have been substantially studied. However, few studies have focused on the combined effects of the seedling-stage DI regime and nutrient application on maize growth and yield formation, as well as water consumption and use. There is still a knowledge gap to understand how nutrient uptake and use in maize plants are affected by DI regime and fertilizer management.

Nitrogen (N) is one of the most important essential nutrients in regulating crop physiological processes and determining grain yield. The coupled N and water managements are prerequisites for sustainable intensive cropping system [17,22,23]. An appropriate N management effectively improved crop photosynthetic capacity by increasing the contents of N, chlorophyll and ribulose-1,5-bisphosphate carboxylase-oxygenase (RuBisCO) and enhancing stomatal conductance (Gs) and transpiration in leaves, consequently increasing dry biomass (DM) accumulation and enhancing plant drought tolerance [24,25]. In addition, sufficient N supply can be helpful by improving the antioxidant enzyme system while maintaining higher osmoprotectants and water contents in plants, thus effectively alleviating the negative influences of drought stress [23,24,26]. In contrast, overuse of N fertilizer was reported to reduce root water absorption and crop transpiration, resulting in greater yield losses induced by the DI regime [15,27]. Plants under higher N input results in higher irrigation water amount to meeting the increased crop water requirement to maintain the balance between water and N uptake in plants [17,28]. Nevertheless, the increased irrigation water might enlarge N leaching into the deeper soil and thus decrease N use efficiency (NUE) while inducing groundwater pollution [21,29]. Previous studies found that crop responses to DI largely depended on fertilizer N supply and management. However, how N fertilization affects the effectiveness of seedling-stage DI on maize seedling growth and whether causes the resulting influences on plant performances during later growing period (e.g., late-vegetative and reproductive growth stages) and final grain yield, WUE and NUE, are still not well understood under field environment with multiple inter-annual climatic conditions under climate changing context.

In this study, a 3-year field experiment was conducted in NEC during maize growing season in 2017, 2018 and 2019, aiming to (1) identify the combined effects of seedling-stage DI and N fertilization on maize seedling growth, plant N uptake and water use, (2) investigate the subsequent effects of seedling-stage DI on plant performance at the silking stage and final grain yield, WUE and NUE at maturity under different N supply conditions and years, and (3) determine the relationships of maize growth performances, water and N use between different growth stages under various soil water and N managements. The findings in this study will be helpful in understanding the N fertilization effects on seedling-stage DI effectiveness in maize production that suffered with increasingly drought stresses under the changing climate and providing useful references for optimizing maize water and N managements in NEC and other regions around the world.

## 2. Results

### 2.1. LAI and Leaf Photosynthetic Parameters after Seedling-Stage DI Period

Soil water level, N fertilization and experimental year showed significant individual effects on LAI and photosynthetic parameters of maize leaves after seedling-stage DI periods (Table 1). Among the 3 years, all the parameters showed consistency in order as 2019 > 2017 > 2018. The seedling-stage DI significantly reduced all the parameters compared with WW plots. The averaged reducing ratio was 9.5% for LAI, 19.7% for Pn, 26.3% for Gs, 18.0% for Ci, 12.4% for Tr and 9.5% for WUE_leaf_, respectively. Among the 3 years, these reductions were generally greater in 2018 while smaller in 2019. However, significant W × Y interaction was only observed for Pn and Gs (Table 1), their reducing ratios due to DI regime in 2018 (28.0% and 34.3%) were considerably higher than those in 2019 (11.2% and 14.3%).

Nitrogen fertilization significantly improved LAI and leaf photosynthetic characteristics regardless of soil water levels and years.The averaged increasing ratio was 23.7%, 43.3%, 22.7%, 15.4%, 11.3% and 29.7% for LAI, Pn, Gs, Ci, Tr and WUE_leaf_, respectively. All the parameters showed greater responses to N fertilization in DI plots compared to WW plots, especially for Pn and WUE_leaf_. The differences of N responses on Pn and WUE_leaf_ depending on soil moisture were much smaller in 2018 compared to the other 2 years. Thus, significant W × N and W × N × Y interactions were observed on Pn and WUE_leaf_ (Table 1). Moreover, compared with WWN1 treatment, DIN1 treatment showed similar performances on LAI in all 3 years and Pn and WUE_leaf_ in 2017 and 2019.

### 2.2. Shoot DM Accumulation at Different Growth Stages

**Figure 1:**
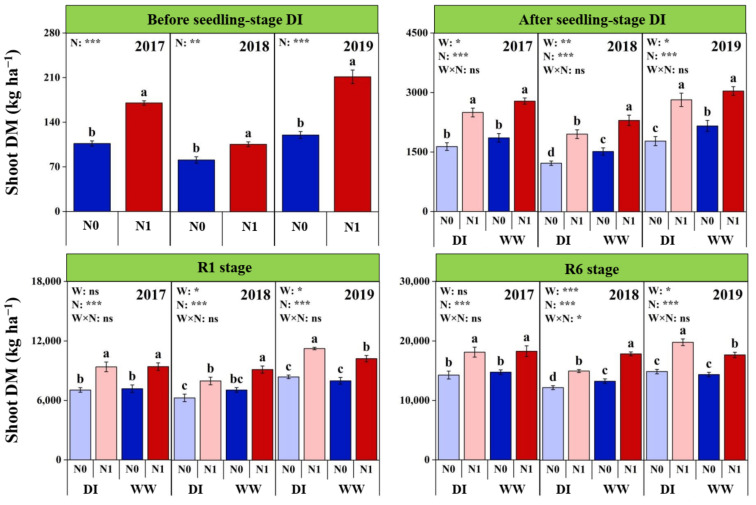
Effects of nitrogen fertilization (N) and seedling-stage water management regime (W) on shoot dry biomass (DM) of maize plants at different growth stages in 2017–2019. Note: *, ** and *** indicate significance at *p* < 0.05, *p* < 0.01 and *p* < 0.001, respectively, and ns indicates non-significance (*p* > 0.05). Different letters above bars indicate significant differences between treatments (*p* < 0.05).

Significant inter-annual differences were observed on shoot DM (SDM) at each growth stage and were generally higher in 2019, followed by 2017, and lowest in 2018 (Figure 1 and Table S1). Particularly, SDM showed greater inter-annual difference at the seedling stage, which were 43.6% and 28.6% lower before and after the DI periods in 2018, respectively, relative to those in 2019. At the R1 and R6 stages, the gaps in SDM between 2018 and 2019 reduced to 19.6% and 12.7%, respectively.

Nitrogen fertilization increased significantly SDM before the seedling-stage DI period, but the increasing ratio was much less in 2018 (30.1%) than those in 2017 and 2019 (76.0% and 59.6%) (Figure 1) Thus, a significant N × Y interaction was observed on SDM at this stage (Table S1). After the DI period, both water and N managements showed significant individual effects on SDM, but no interaction was detected (Figure 1 and Table S1). The DI significantly reduced SDM with an averaged reduction of 12.8% across the 3 years; N fertilization significantly increased SDM. The averaged increasing ratio was higher in DI plots (56.7%) than WW plots (46.8%). In spite of the higher increasing ratio with N fertilization, SDM were still lower in DIN1 treatment relative to WWN1 treatment, and the difference was significant between treatments in 2018.

At the R1 and R6 stages, seedling-stage DI showed different effects on SDM among the 3 growing seasons. Thus, significant W × Y interactions were detected (Figure 1 and Table S1). Compared with WW plots, SDM in DI plots decreased by 12.0% and 12.7% at the R1 and R6 stages in 2018, respectively. However, the opposite was observed by an increase of 7.8% and 8.2% in 2019, respectively, and no differences were observed between soil water levels in 2017. Shoot DM increased significantly with N fertilization at the R1 and R6 stages. The averaged increasing ratios were 30.5% and 27.5%, respectively. Compared with WWN1 treatment, SDM in DIN1 treatment was higher in 2019 but lower in 2018, and no difference was observed in 2017.

### 2.3. Root DM Accumulation at Different Growth Stages

**Figure 2:**
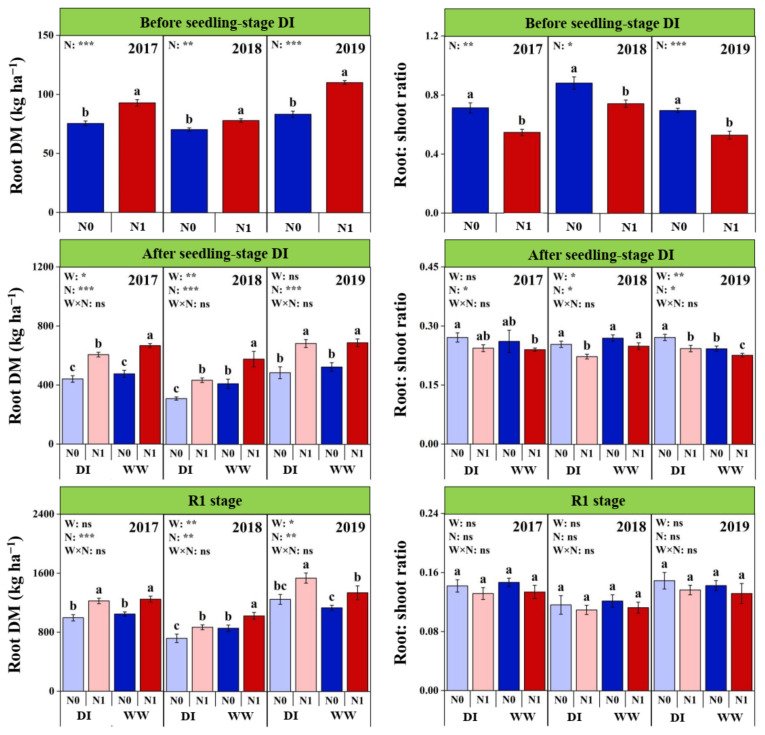
Effects of nitrogen fertilization (N) and seedling-stage water management regime (W) on root dry biomass (DM) and root: shoot ratio of maize plants at different growth stages in 2017–2019. Note: *, ** and *** indicate significance at *p* < 0.05, *p* < 0.01 and *p* < 0.001, respectively, and ns indicates non-significance (*p* > 0.05). Different letters above bars indicate significant differences between treatments (*p* < 0.05).

Similar with SDM, root DM (RDM) showed significant inter-annual differences during the three growing seasons, and also followed the consistent order as 2019 > 2017 > 2018 (Figure 2 and Table S1). However, RDM in 2018 were 23.3%, 27.2% and 33.9% lower at the BDI, ADI and R1 stages relative to 2019, respectively, showing an increasing gap in RDM between years over time. The significant W × Y interactions were observed on RDM at both the ADI and R1 stages (Figure 2 and Table S1). Compared with WW plots, DI significantly reduced RDM at the ADI stage in both 2017 and 2018 but had no difference in 2019; at the R1 stage, RDM in DI plots was higher significantly in 2019 while significantly lower in 2018, but equal in 2017. Nitrogen fertilization significantly increased RDM at each growth period (Figure 2 and Table S1). At the BDI stage, a significant N × Y interaction was also observed on RDM. Its increasing ratio with N fertilization was the highest in 2019 (32.2%), followed by 2017 (23.1%), and was the lowest in 2018 (10.9%). At the ADI and R1 stages, N fertilization increased RDM by 38.2% and 20.7%, respectively. Compared with WWN1 treatment, RDM in DIN1 treatment were lower at the ADI stage in 2017 and 2018, as well as the R1 stage in 2018, but which was higher at the R1 stage in 2019.

Significant inter-annual differences were observed on the root: shoot ratio at both the BDI and R1 stages, but not at the ADI stage (Figure 2 and Table S1). Compared with other years, the root: shoot ratio in 2018 was higher at the BDI stage while lower at the R1 stage. At the ADI stage, the root: shoot ratio was significantly affected by W × Y interaction, which decreased by 8.1% in 2018 but increased by 9.8% in 2019 with implementing the DI regime and did not change in 2017. In each year, N fertilization significantly reduced root: shoot ratio at the seedling stage, the reducing ratios were 20.6% and 9.2% before and after the DI period, respectively. At the ADI stage, the root: shoot ratio in DIN1 treatment was higher in 2019 but lower in 2018, relative to WWN1 treatment. At the R1 stage, although the effects were not statistically significant, the root: shoot ratio tended to decrease with N fertilization, meanwhile showed increasing trend under the seedling-stage DI in 2019.

### 2.4. Grain Yield and Components at Maturity

**Figure 3:**
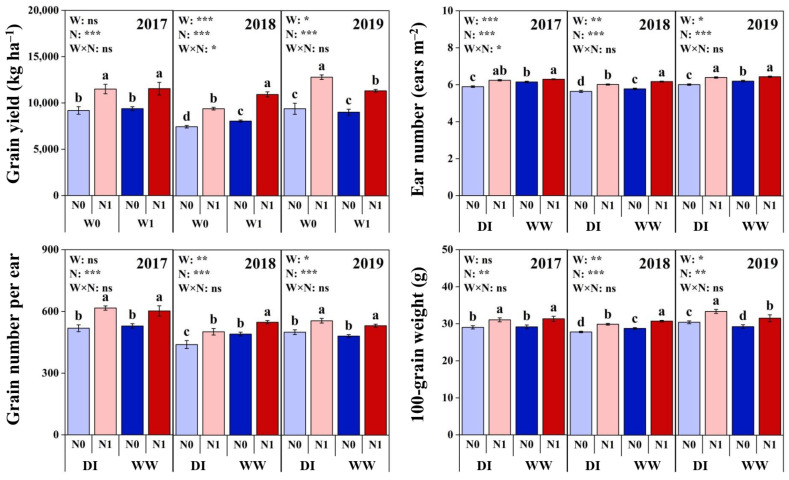
Effects of nitrogen fertilization (N) and seedling-stage water management regime (W) on maize grain yield and yield components at maturity in 2017–2019. Note: *, ** and *** indicate significance at *p* < 0.05, *p* < 0.01 and *p* < 0.001, respectively, and ns indicates non-significance (*p* > 0.05). Different letters above bars indicate significant differences between treatments (*p* < 0.05).

In the period of technological maturity, the grain yield and all yield components were significantly different depending on the year of the study (Figure 3 and Table S1). The highest grain yield, ear number and 100-grain weight were observed in 2019. The highest grain number per ear was found in 2017, while all the lowest values were obtained in 2018. The final ear number at maturity decreased significantly due to seedling-stage DI in each year. Grain yield, grain number per ear and 100-grain weight showed different responses to DI among the 3 growing seasons. Compared with WW plots, the three parameters in DI plots were lower in 2018 while higher in 2019, and no differences were observed in 2017.

Nitrogen fertilization significantly increased grain yield and yield components across different soil water levels and years and showed W × N and N × Y interactions on ear number (Figure 3 and Table S1). Between two soil water levels, ear number showed greater response to N fertilization in DI plots as compared to WW plots. Among the 3 years, the N response of ear number was greater in 2018 than other years. Moreover, compared with WWN1 treatment, all the parameters in DIN1 treatment were lower in 2018, while grain yield and 100-grain weight were higher in 2019, but with no differences in 2017.

### 2.5. Plant N Uptake and NUE at Different Growth Stages

**Figure 4:**
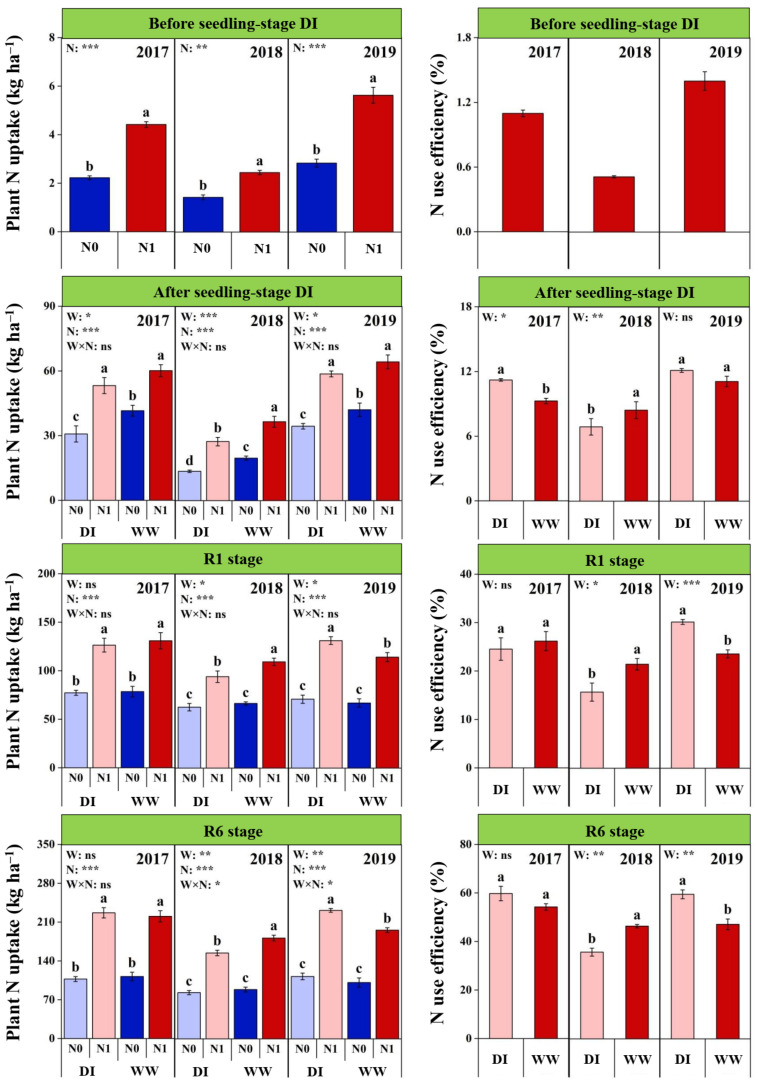
Effects of nitrogen fertilization (N) and seedling-stage water management regime (W) on N uptake and N use efficiency of maize plants at different growth stages in 2017–2019. Note: *, ** and *** indicate significance at *p* < 0.05, *p* < 0.01 and *p* < 0.001, respectively, and ns indicates non-significance (*p* > 0.05). Different letters above bars indicate significant differences between treatments (*p* < 0.05).

The N uptake (NU) and NUE of maize plants showed significant inter-annual differences at each growth stage, both of which were lower in 2018 than other years (Figure 4 and Table S2). Nitrogen fertilization increased NU in each year, and the averaged increasing ratios were 92.7% before DI, 64.8% after DI, 67.0% and 100.1% at the R1 and R6 stages, respectively. In addition, NU was affected by the N × Y interaction at the BDI, R1 and R6 stages, while its N responses were significantly lower in 2018 than other years. The DI significantly reduced NU at the seedling stage with an averaged reduction of 17.5% across the 3 years. Nevertheless, NU was affected by the W × N × Y interaction at both the R1 and R6 stages, which was equal between DIN0 and WWN0 treatments in each year, but which for DIN1 treatment was lower in 2018 while higher in 2019 relative to WWN1 treatment. The NUE was significantly affected by seedling-stage DI during the entire growing season, but the responses differed among the 3 years (Figure 4 and Table S2). Compared with WW plots, the DI significantly decreased NUE at each stage in 2018, but significantly increased it at the ADI stage in 2017 as well as the R1 and R6 stages in 2019.

### 2.6. ETa and WUE during Different Growth Periods

**Figure 5:**
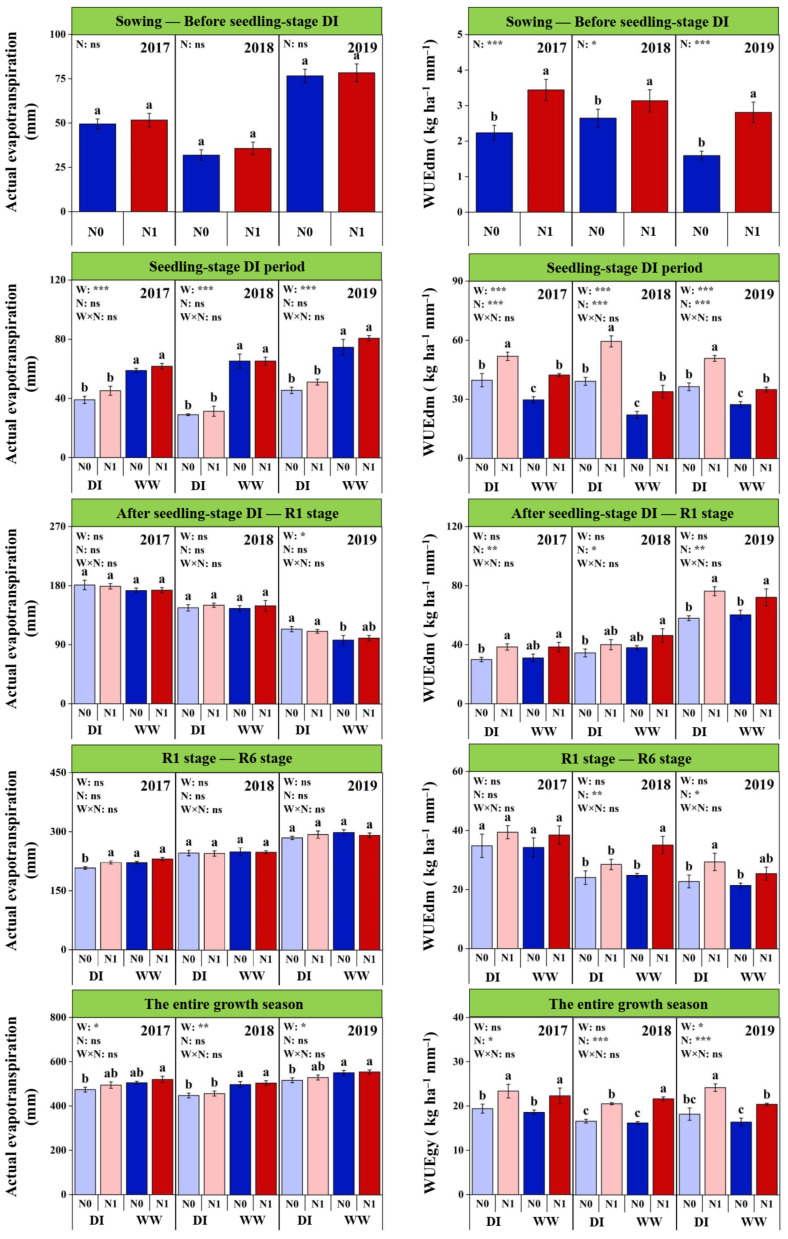
Effects of nitrogen fertilization (N) and seedling-stage water management regime (W) on actual evapotranspiration and water use efficiency for dry biomass (WUEdm) at different growth periods and grain yield (WUEgy) in 2017–2019. Note: *, ** and *** indicate significance at *p* < 0.05, *p* < 0.01 and *p* < 0.001, respectively, and ns indicates non-significance (*p* > 0.05). Different letters above bars indicate significant differences between treatments (*p* < 0.05).

Significant inter-annual differences were observed on both ETa and WUEdm at all growth periods, except for the WUEdm during the seedling-stage DI period (Figure 5 and Table S2). During the ADI-R1 period, the lowest ETa and the highest WUEdm were obtained in 2019; however, at the other periods, both the highest ETa and the lowest WUEdm were found in 2019. Except for the ADI-R1 period, the highest WUEdm was observed in 2017 during the entire growing season. Among the 3 years, the total ETa followed the order as 2019 (538.5 mm) > 2017 (498.6 mm) > 2018 (477.3 mm), while the order of WUEgy was 2017 (20.9 kg ha^−1^ mm^−1^) > 2019 (19.8 kg ha^−1^ mm^−1^) > 2018 (18.8 kg ha^−1^ mm^−1^).

The DI significantly reduced the ETa at the seedling period, but the reduction relative to WW plots showed significant differences among the 3 years, which was considerably higher in 2018 (53.6%) than 2017 and 2019 (30.1% and 37.7%) (Figure 5 and Table S2). The seedling-stage DI trended to increase the ETa at ADI-R1 period across all the 3 years, but significant increment was only observed in 2019. Compared with WW plots, total ETa in DI plots were 5.5%, 9.7% and 5.4% lower in 2017–2019, respectively. With regard to WUEdm, it was significantly increased under the DI regime at the seedling stage in each year, but the increasing ratio was considerably higher in 2018 (75.8%) than 2017 and 2019 (27.0% and 39.8%). In contrast, the final WUEgy was only improved significantly with the seedling-stage DI regime in 2019 (15.1%), while it showed no significant differences in other years.

Nitrogen fertilization tended to increase the ETa before the seedling-stage DI period but showed no significant effects on the ETa throughout entire growing season (Figure 5). In contrast, N fertilization improved significantly the WUEdm at each period and final WUEgy across the 3 years, except for the R1-R6 period in 2017 (Figure 5 and Table S2). On average, the WUEgy was increased by 25.6% with N fertilization across the soil water levels and years. Compared with WWN1 treatment, the WUEgy in DIN1 treatment was lower in 2018, higher in 2019 while equal in 2017.

### 2.7. Relationships among Maize Growth, Water and N Use Parameters at Different Periods

**Figure 6:**
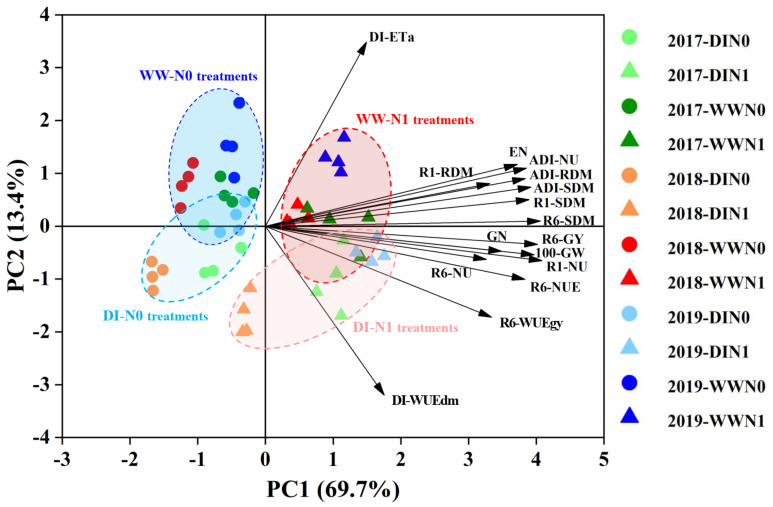
Principal component analysis (PCA) biplot of maize growth and water and N use parameters at different periods under various nitrogen and seedling-stage soil water managements in 2017–2019. Note: DI, deficit irrigation; WW, well-watered irrigation; N0, N-omission; N1, sufficient N supply; ADI, after DI period; R1, silking stage; R6, maturity stage; ETa, actual evapotranspiration; SDM, shoot dry matter; RDM, root dry matter; NU, plant N uptake; NUE, N use efficiency; GY, grain yield; EN, ear number; GN, grain number per ear; 100-GW, 100-grain weight; WUEdm and WUEgy, water use efficiency for SDM and GY, respectively.

To explore the overall relationships within the important parameters of maize growth, water and N use at the different periods and their responses to N fertilization and seedling-stage DI, both the principal component analysis (PCA) and correlation analysis were conducted in this study (Figure 6 and Figure S1). The PCA results showed that 83.1% of the total variability was explained by the first two principal components (PC). The PC1 explained 69.7% of the variability, and mainly accounted for the SDM, RDM and NU at different periods, and grain yield, yield components, WUEgy and NUE at maturity, while PC2 represented 13.4% of variability mainly derived from the ETa and WUEdm during the DI period. Clearly, a significant and negative correlation (r = −0.61 ***) was observed between DI-ETa and DI-WUEdm, while the correlations were significant and positive between all the other parameters. The DI-ETa showed stronger positive correlations with most of the growth parameters at the ADI and R1 stages, as well as EN at the R6 stage; in contrast, the DI-WUEdm represented stronger positive correlations with most of those parameters at the R6 stage and NU at the R1 stage.

The PCA allowed clear discrimination among the treatments with different soil water and N managements in the biplot (Figure 6). The clusters of N treatments projections were totally separated along the PC1, those marked with blue or red colors linked to N0 or N1 treatments, respectively. Regardless of soil water regimes, most of the projections for N1 treatments were clustered toward the vector direction as plant growth and yield parameters, except for DIN1 treatment in 2018. As for soil water regimes, a partly overlapped distinction was observed between WW and DI projection clusters along the PC2. Compared with both WWN0 and DIN0 treatments, most of the WW-N1 projections were positively related to higher DI-ETa and better growth performances at the ADI and R1 stages, in contrast, most of those for DIN1 treatments were positively associated with higher DI-WUEdm and better performances for NUE and WUEgy at maturity. In addition, the projections in 2017 located closer to the center of PC2, indicating that soil water and N managements had relatively less effects on these parameters in 2017 as compared with those in 2018 and 2019.

## 3. Discussion

### 3.1. Maize Seedling Growth Responses to DI Regime in Different Years

The seedling stage is a key period for determining maize plant development and root system architecture, and which were greatly affected by soil water management [9,30,31]. Previous studies have shown the various water deficit levels by regulating water supply as 40%–80% of the total ETa during maize growing season, decreased significantly LAI, leaf chlorophyll content and several key photosynthetic indexes for maize plants, resulted in plant growth limitation and yield reduction, as compared to the full ET irrigation [18,32]. In this study, the significant reductions were similarly observed both for SDM and RDM for maize seedlings exposed to DI regime across the three growing seasons, compared to WW plants. The reduced plant DM accumulation under the DI regime was synchronously associated with decreased LAI and generally down-regulated photosynthesis parameters. Several reports emphasized that the negative effects of the DI regime depend on maize growth period and the duration and severity of water stress [9,10,14]. At the seedling stage, the down-regulated LAI and photosynthesis induced by moderate and temporary water deficit may not result in permanent injury for maize leaf growth and function, which can largely recover with re-watering and even show better growth performance. Meanwhile, an appropriate water deficit can increase the allocation of photosynthetic assimilates into root system to promote root elongation and distribution in deeper soil, and further enhance water capture and plant adaption to water stress [30,31,33]. In contrast, the irreversible damage of maize seedlings induced by severe water deficit can impact reproductive growth and even grain yield [15,16,18]. In other words, an appropriate and short-term DI regime adopted at the seedling stage is beneficial for controlling the redundant vegetative growth for maize plants while optimizing root architecture, therefore effectively improving plant tolerance to drought stress and WUE [9,11].

**Figure 7:**
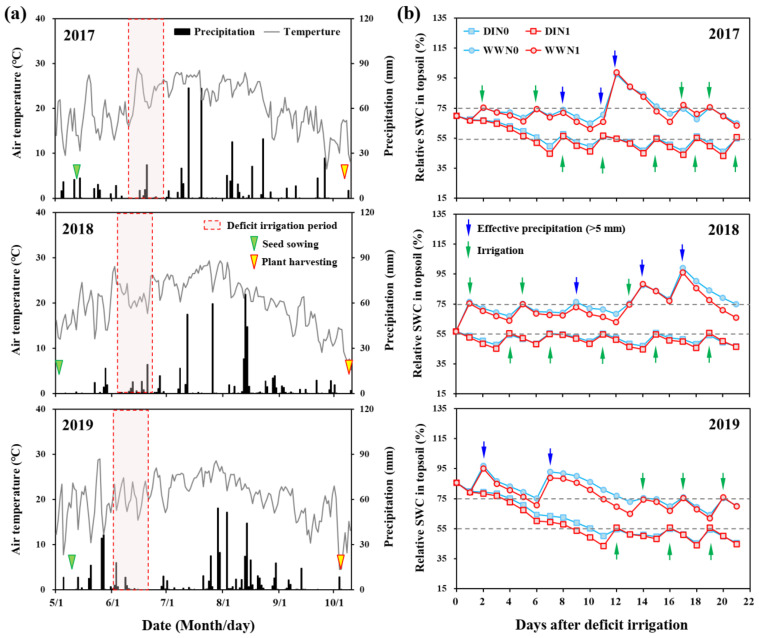
Daily precipitation (bars) and average air temperature (line) throughout the entire maize growing season (**a**) and dynamic of relative soil water content (SWC) in topsoil (0–20 cm) during the seedling-stage deficit irrigation periods (**b**) in 2017–2019 at Lishu county, Northeast China.

In this study, a greater negative effect of the DI regime was observed on maize growth in 2018 relative to other years, which was mainly attributed to the contrasting inter-annual climatic conditions before implementing the DI regime. In 2018, less precipitation during sowing to early-seedling period led to a lower topsoil SWC (57% of FC) and a relatively weaker seedling growth before the DI period (Figure 7). In this case, the DI regime adopting with a moderate water deficit level aggravated the pre-existing water stress and seriously limited plant growth [34,35], thus resulting in 24.7% and 16.8% of reductions for SDM and RDM, respectively. In addition, the root: shoot ratio also decreased with the DI regime in 2018, indicating that the insufficient photosynthetic products could not provide additional support for root growth [30,36]. Moreover, the server water stress under the DI regime in 2018 reduced soil N availability, especially for limiting nitrate movement and capture by roots. Therefore, the poor root growth and lower soil N availability resulted in a greater decrease on N uptake for maize plants under DI regime in 2018 relative to other years [17,37]. In 2017 and 2019, the higher initial SWC ensured well seedling growth, thereby providing a firm foundation to adopt the DI regime and helping plants to train and adapt to water stress. Therefore, interestingly during these two growing seasons, the equal or higher root: shoot ratio was obtained in DI plots relative to WW plots, by controlling overgrowth for shoot while enhancing root development. Our results suggest that the positive effects of the DI regime at the maize seedling stage requires flexibly and timely adjusted water management in regard to climatic condition and SWC status.

### 3.2. Effects of N Fertilization on the Seedling-Stage DI Effectiveness

As another important factor that affected crop growth and yield formation, fertilizer N application and management also received much attention in intensive maize production around the world [38,39]. Some scholars have investigated the effects of different N rates, fertilization time and fertilizer types on maize growth, grain yield and WUE under the varying DI regimes [15,16,21], and also evaluated and determined the optimal DI and N management strategies [17]. However, there is limited information about the combined effects of N fertilization and seedling-stage DI regime on maize growth, water and N use efficiencies under field conditions with diverse climates. In this study, regardless of different water regimes and years, N fertilization increased significantly plant DM accumulation and N uptake at the seedling stage. Moreover, maize seedling growth showed greater responses to N fertilization under the DI regime relative to WW condition, suggesting that N fertilization had a compensatory effect on maize growth under water stress. This compensatory effect of N fertilization was also supported by previous studies, the increased soil N amount and availability with N fertilizer applied effectively alleviated N deficiency in plant associated with water stress [16,25,40]. Therefore, the N-treated plants exposed to the DI regime showed higher LAI and leaf photosynthetic capacity, and increased significantly DM accumulation compared to the untreated plants. Moreover, N fertilization effectively enhanced plant drought tolerance, which was partly attributed to the improved cell osmotic potential by producing more osmoprotectants including soluble proteins, soluble carbohydrates and free proline, and also associated with the enhanced redox defense status by enhancing antioxidant system and reducing MDA accumulation [17,20,23,26].

The significant inter-annual differences were found on the combined effects of N fertilization and the DI regime on maize seedling growth and N uptake. In 2017 and 2019, N fertilization played greater compensatory effects under the DI regime, narrowing the gap in seedling growth between DI and WW plots. Compared with WWN1 treatment, the DIN1 treatment showed equal plant DM accumulation and N uptake and higher NUE in these 2 years. In contrast, a less compensatory effect of N fertilization to water stress under DI regime was obtained in 2018, due to the lower precipitation during seedling period [17,41]. Similar with the previous studies [32,37,40], although N fertilization increased both Tr in leaf and the ETa for maize seedling in this study, it played substantial positive effects on improving Pn and SDM and thus resulted in higher WUE in both leaf and whole plant scales. Compared to the WW plants, N fertilization dramatically increased WUE for plants grown under the DI regime, due to reduced water supply and subsequently lower soil evaporation. In addition, between DIN1 and WWN1 treatments, the WUE improvements in 2018 was considerably greater than those in 2017 and 2019.

### 3.3. Combined Effects of N Fertilization and Seedling-Stage DI on Later Plant Performances

In this study, maize plant performances at the R1 stage and grain yield, WUE and NUE at maturity were strongly related to the growth status at the seedling stage which was affected by soil water and N managements. The corresponding results were illustrated clearly in the PCA and correlation analysis. In addition, the quantity and distribution of precipitations led to contrasting patterns of soil water supply during the later growth periods among the 3 years. The rainless climate during July induced severe soil drying before maize silking in 2019, whereas heavy rainfall occurred around the silking stage in both 2017 and 2018. All these climatic adversities potentially limited floret development and pollination at the R1 stage, as well as the subsequent kernel formation and grain filling [18,22,42]. Therefore, significant inter-annual differences were also observed on maize plant performances at later growth period, due to various climatic conditions and seedling growth status. In 2017, whether N fertilization or not, the primed plants under the DI regime showed equal growth and yield performances compared to WW plants at the R1 and R6 stages, and also tended to increase NUE and WUEgy although the increments were statistically insignificant. In 2019, the vigorous seedling growth, higher root: shoot ratio and enhanced drought tolerance induced by DI regime at seedling stage made maize plants better adapt to the subsequent drought stress [24,43,44], and ensured well plants growth at the R1 stage and satisfactory yield level similar to WW plants. In generally, the comparable grain yield, plant N uptake and WUEgy were observed between DIN0 and WWN0 treatments. In N fertilization treatments, the primed plants in DIN1 showed superior silking growth and higher grain yields compared to the WWN1 plants, by maintaining higher radiation interception with larger leaf area, and extending green leaf duration and enhancing photosynthesis [20,32]. This suggested that seedling-stage DI regime and N fertilization had positive combined effects on plant performances and yield formation, as well as the capture and use for water and N resources. However, in 2018, the plants under the seedling-stage DI regime did not recover to similar growth level as WW plants until the R1 stage, showing worse growth performances even under N fertilization. The weak seedling growth and root development limited ear formation in part of plants under the DI regime and significantly reduced effective plants for harvesting, meanwhile restricted ear development and grain filling. This made it more difficult to produce ideal ear structures at similar number and size as those WW plants, eventually caused significant yield losses and lower NUE and WUE.

Maize plant growth status at the R1 stage and subsequent grain-filling process are of vital importance for determining yield formation, and if drought stresses occur around this period often result in higher yield losses compared to other growth stages [18,21]. How to mitigate the negative effects of drought stress during reproductive growth period is a hot research topic for maize production in arid and semiarid regions [3,42,45]. Based on our results in 2019, implementing the seedling-stage DI regime was proven as a promising approach to help maize seedling to enhance stress tolerance. Plants were also shown to possess stronger adaption capacity to subsequent drought stresses during reproductive growth period, and synergistically achieving higher grain yield and WUEgy. Li et al. [46] have reported that irrigation and fertilization practices contributed 20.6% and 32.8% to the WUE increase for irrigation water, respectively, in the Hexi Corridor region, Northwest China. It is suggested to appropriately optimize soil water and N managements to play their coupled effects on maize production, for dealing with the changing climate and improving crop productively and WUE. According to the results in this study, the full irrigation is unnecessary for maize at the seedling stage in NEC, because of less water demand from young plants, and most of water are lost through soil evaporation rather than plant transpiration due to the incomplete canopy closure [45]. Instead, a moderate DI regime should be adopted based on the initial SWC condition, to achieve a better effectiveness for saving water resource and enhancing plant potential capacity to resistance to water stress. Under the DI regime, N fertilization is helpful to increase leaf area and thereby reducing soil evaporation and maintaining higher residual water in soil, whereas could also improve root growth and enhance water capture and use in plants. Thus, N supply should be properly managed and matched with the DI regime during seedling stage, to maintain a balance between water and N availability and effectively play its compensatory effects to water stress. In addition, the PCA results indicated that N fertilization had greater effects on maize plant growth and the use for N and water resources relative to the DI regime throughout the entire growing season. This is possible associated with the relatively shorter duration of DI at the seedling stage and suggested that the seedling-stage DI regime is required to adopt with proper N management method.

## 4. Materials and Methods

### 4.1. Experiment Site Description

A field experiment was conducted over 3 maize growing seasons during 2017 to 2019 at the experiment station of Jilin Agricultural University, located at Lishu County, Jilin Province, NEC (43°20′16′′ N, 124°03′36′′ E). The experimental field has loam soil with a 1.41 g cm^−3^ bulk density, 0.288 cm^−3^ cm^−3^ field capacity (FC), 5.14 pH, 29.4 g kg^−1^ organic matter (dichromate oxidation method), 126.2 mg kg^−1^ available N (alkaline hydrolysable method), 39.6 mg kg^−1^ Olsen-P, and 130.7 mg kg^−1^ NH_4_OAc-K, with 47.7% sand, 29.6% silt and 22.7% clay in the 0–20 cm soil layer. The soil is classified as Alluvic Primosol according to Chinese soil taxonomy, and Fluvisols according to the World Reference Base soil classification system. The experimental site experiences a semi-humid continental monsoon climate with an average precipitation of 570 mm year^−1^ and an average annual temperature of 5.8 °C. The precipitation during maize growing season was 491, 471 and 534 mm in 2017, 2018, and 2019, respectively. However, considerable inter-annual precipitation difference was observed in May among the 3 years, which was 67, 36 and 115 mm, respectively. In addition, a severe drought occurred during June and July in 2019, when was the period for maize later-vegetative growth and silking. The detailed climatic information during the 3 growing seasons is shown in Figure 7a.

### 4.2. Experimental Design and Field Management

The experimental treatments included two N-fertilization rates and two soil water management regimes at the seedling stage, which were laid out as a randomized block region with four replications. The two N rates included 0 and 200 kg N ha^−1^, representing N-omission (N0) and sufficient N supply (N1), respectively. In addition to N fertilization, 39.3 kg P ha^−1^ and 75 kg K ha^−1^ were applied to meet the nutrient requirement for successful maize plant growth. The basal fertilizer, urea (46% N), triple super-phosphate (20% P) and muriate of potash (50% K) were once applied and used as fertilizer resources. The water management regimes at the seedling stage were initiated from the V3 stage and lasted for 3 weeks to about the V6–V7 stage (Figure 7a), including two water levels with different relative soil water content (SWC) in 0–20 cm topsoil those controlled as 55 ± 5% (DI regime) and 75 ± 5% (WW regime, i.e., well-watered by supplementing irrigation on the basis of natural precipitation) of FC, respectively.

The size of each plot was 30 m^2^ (6 × 5 m), and maize cultivar Liangyu 99 was sown by using a flat planting method, with an identical density of 65,000 plants ha^−1^ across all the plots in each year. The sowing dates were 11th, 2rd and 9th May in 2017, 2018 and 2019, respectively, and the plants were harvested on 7th, 9th and 5th October in the 3 years, respectively (Figure 7a). During seedling-stage DI period, a large mobile rain-shelter was used to cover the DI plots only when it rained, whereas the WW plots were exposed to the air and received natural rainfall. The supplemental irrigation was applied when the relative SWC in topsoil declined to the predetermined lower limits in DI and WW plots (i.e., 50% and 70% of FC, respectively), and irrigation amount was calculated according to the difference between predetermined and actual SWC. The micro-sprinkling hose method was used for irrigating in this study, because of the significant advantages in improving uniformity of irrigation while reducing input and complexity of irrigation facilities relative to conventional drip and sprinkler irrigation [47]. In each plot, irrigation was performed using a pump coupled with a flow-meter, and water amount was recorded for calculating water balance in soil-plant system. The dynamic of relative SWC in topsoil and the times of supplemental irrigation and effective precipitation during 3-week DI periods in the 3 years are shown in Figure 7b. The topsoil SWC in DI plots reached the predetermined values at 6, 1 and 10 days after implementing DI regime in 2017–2019, respectively. The variable durations for soil drying were mainly related to the different inter-annual SWC in topsoil before DI periods, which were 69.8%, 56.7% and 85.5% of FC in three years, respectively. In addition to seedling-stage DI periods, no irrigation was conducted in all the plots during rest of the growing season. Other management practices including soil tillage and the control of weed, diseases and pests were conducted using local recommended methods.

### 4.3. Sampling and Measurement

The volumetric SWC (vol%) in 0–100 cm soil profile was measured in each plot using the time-domain reflectometer (TRIME-PICO IPH, IMKO, Ettlingen, Germany) with a 20-cm-long probe. Three PVC tubes (a 100 cm long PVC tube with a 44 mm outer diameter and 42 mm inner diameter) were installed in three maize rows for each plot by using an auger, and the three-point average within a same soil layer represented SWC in each plot. The SWC was measured every day during the seedling-stage DI period, as well as the days at sowing, the R1 and R6 stages. The SWC in 0–20 cm topsoil measured during DI period was used for controlling soil water levels required by different irrigation regimes, and the SWC in 0–100 cm soil profile measured at different stages were used for calculating the water balance in soil-plant system.

Leaf area index (LAI) and leaf photosynthetic parameters were measured 1 or 2 days before ending seedling-stage DI regime in all the 3 growing seasons. In each plot, the leaf length and width were measured for all leaves from ten representative plants to calculate the LAI. Thereafter, first fully expanded leaf from top of the ten maize plants were selected for measuring the net photosynthetic rate (Pn), stomatal conductance (Gs), intercellular CO_2_ concentration (Ci) and transpiration rate (Tr) by using a LI-6800 photosynthesis system (LI-COR, Lincoln, NE, USA) in each plot, with a controlled light density of 1500 μmol m^−2^ s^−1^ and the CO_2_ concentration in leaf chamber of 400 μmol CO_2_ (mol air)^−1^ between 9:00 and 11:00 am under a clear sky. The instantaneous water use efficiency in leaf (WUE_leaf_) was calculated as following:

WUE_leaf_ = Pn/Tr (1)

Five successive and representative plants from the central rows of each plot were sampled before and after the seedling-stage DI periods (i.e., BDI and ADI stages, respectively), as well as the R1 and R6 stages, then root system of three sampled plants were also excavated at the BDI, ADI and R1 stages. Shoot and root samples were first oven-dried at 105 °C for 30 min and then at 70 °C until a constant weight to obtain their DM (i.e., SDM and RDM, respectively). The N concentrations in shoot samples at different growth stages were analyzed by using Kjeldahl method, and then to calculate plant N uptake (NU) and NUE according to the equations described by Dobermann (2007) [48]. At the R6 stage, ear number and grain yield were determined by harvesting an area of 12 m^2^ in each plot. Grains were oven-dried to determine the moisture content, and grain yield was standardized to 14% moisture. Ten representative plants were selected from the harvesting area in each plot to determine grain number per ear and 100-grain weight.

The actual evapotranspiration (ETa, mm) in soil-plant system was calculated according to the soil water balance equation [49]:

ETa  =  P + I + Cr − R − D ± ΔSWC(2)

where P is precipitation, I is irrigation water, Cr is capillary rise, R is run off, D is drainage water below the 100 cm soil layer, and ΔSWC is the changed SWC in 0–100 cm soil profile. Because the soil surface was flat and the groundwater table was 10 m below soil surface, the R and Cr were considered negligible. It was assumed that no drainage occurred below 100 cm soil layer according to Sun and Li (2019) [50].

The water use efficiency for grain yield (WUEgy) and shoot DM (WUEdm) were calculated as following [51]:

WUE_gy_ = grain yield/ETa (3)

WUE_dmn_ = Shoot DM_n_/ETa_n_ (4)

where ETa is the total ETa throughout the maize entire growing season. In order to identify the effect of seedling-stage DI and N fertilization on plant water consumption and WUE at different growth periods, the ETa and Shoot DM were divided into four periods (i.e., ETa_n_ and Shoot DM_n_, n = 1, 2, 3, 4, respectively, indicating S–BDI, BDI–ADI, ADI–R1 and R1–R6, respectively). Correspondingly, the WUEdm_n_ was the ratio of Shoot DM_n_ to ETa_n_ at a specific growth period.

### 4.4. Statistical Analyses

After verifying the homogeneity of error variance, all the data across soil water regimes, N rates and years were examined by analysis of variance (ANOVA) using SPSS 18.0 (SPSS Inc., Chicago, IL, USA). Differences were compared using the significant difference test (LSD) at the 0.05 level of probability. A principal component analysis (PCA) and a Pearson correlation analysis were conducted to reveal the relationships within the selected parameters (including ETa and WUEdm during seedling-stage DI period, SDM, RDM and NU at different stages, grain yield and yield components, WUEgy and NUE at maturity) under the different soil water and N managements using the OriginPro 2021 (OriginLab, North-hampton, MA, USA).

## 5. Conclusions

During the 3 experimental years in this study, seedling-stage DI regime generally limited leaf area and photosynthesis and reduced shoot DM for maize seedling under field environment, and N fertilization showed significant compensatory effects to water stress under DI regime. However, seedling-stage DI and N fertilization showed different combined effects on seedling growth, as well as silking growth and final grain yield, due to the various climatic conditions during early-vegetative growth period among 3 years. Under sufficient rainfall and higher initial field SWC, a short-term and moderate seedling-stage DI regime is suggested to couple with appropriate N fertilization to control redundant vegetative growth while optimizing root development, to enhance plant drought tolerance and allow to produce a better reproductive growth, and consequently achieve higher grain yields, WUE and NUE. The results in this study have important implications for improving DI regime and N management for maize production in NEC and other regions where are facing increasing drought stresses under changing climate.

## Figures and Tables

**Table 1:** Effects of N fertilization and seedling-stage deficit irrigation (DI) on leaf area index (LAI) and net photosynthetic rate (Pn), stomatal conductance (Gs), intercellular CO_2_ concentration (Ci), transpiration rate (Tr) and leaf instantaneous water use efficiency (WUE_leaf_) of maize plant after DI period in 2017–2019.

Year	Treatment	LAL	Pn	Gs	C_i_	Tr	WUE_leaf_
			μmol CO_2_ m^−2^ s^−1^	mol H_2_O m^−2^ s^−1^	μmol CO_2_ mol^−1^	mmol H_2_O m^−2^ s^−1^	μmol CO_2_ mmol^−1^ H_2_O
2017	DIN0	2.1 b	21.8 c	0.20 c	78.3 c	5.0 c	4.4 c
	DIN1	2.6 a	37.9 ab	0.26 b	90.4 b	5.6 b	6.8 a
	WWN0	2.3 b	33.9 b	0.30 b	95.8 b	5.7 b	5.9 b
	WWN1	2.8 a	41.9 a	0.36 a	107.0 a	6.1 a	6.8 a
2018	DIN0	1.7 c	18.2 c	0.16 c	62.8 c	4.2 c	4.4 c
	DIN1	2.3 ab	28.0 b	0.23 b	76.2 b	5.2 b	5.4 b
	WWN0	2.1 b	25.7 b	0.27 b	85.4 b	5.4 b	4.8 c
	WWN1	2.5 a	38.5 a	0.34 a	97.7 a	6.0 a	6.4 a
2019	DIN0	2.2 b	27.0 c	0.28 b	82.0 c	5.4 c	5.0 c
	DIN1	2.7 a	42.8 a	0.31 b	96.1 b	5.9 b	7.2 a
	WWN0	2.4 b	35.4 b	0.32 b	96.5 b	6.0 b	5.9 b
	WWN1	2.9 a	43.2 a	0.37 a	110.4 a	6.4 a	6.8 a
**Source of variation**							
Water (W)		***	***	***	***	***	***
Nitrogen (N)		***	***	***	***	***	***
Year (Y)		***	***	***	***	***	***
W × N		ns	**	ns	ns	ns	*
W × Y		ns	*	*	ns	ns	ns
N × Y		ns	ns	ns	ns	ns	ns
W × N × Y		ns	**	ns	ns	ns	**

Note: *, ** and *** indicate significance at *p* < 0.05, *p* < 0.01 and *p* < 0.001, respectively, and ns indicates non-significance (*p* > 0.05). Means followed different letters in each year indicate significant differences between treatments (*p* < 0.05).

## Data Availability

The data presented in this study are available on request from the corresponding author.

## Supplementary Materials

The following supporting information can be downloaded at https://www.mdpi.com/article/10.3390/plants11213007/s1. Table S1: Summary of ANOVA for the effects of seedling-stage soil water level, N fertilization and experimental year on shoot and root dry biomass (DM) and their ratio at different growth stages, grain yield and yield components of maize plants; Table S2: Summary of ANOVA for the effects of seedling-stage soil water level, N fertilization and experimental year on N uptake, N use efficiency (NUE), actual evapotranspiration (ETa) and water use efficiency (WUE) for shoot dry biomass (WUEdm) and grain yield (WUEgy) of maize plants at different growth stages; Figure S1 Correlations coefficients (upper triangular) and significant marks (lower triangular) among maize growth, water and N use parameters at different periods, respectively.
