# Supplementary material for: Seedling-Stage Deficit Irrigation with Nitrogen Application in Three-Year Field Study Provides Guidance for Improving Maize Yield, Water and Nitrogen Use Efficiencies
Source: Plants (Basel). 2022 Nov 7;11(21):3007. doi: 10.3390/plants11213007 (PMC9656380; doi:10.3390/plants11213007)
Supplement: Supplementary file 1 [file plants-11-03007-s001.zip › plants-1990626-supplementary.pdf]

Supplementary Materials

Seedling-Stage Deficit Irrigation with Nitrogen Application in Three-Year Field Study Provides Guidance for Improving Maize Yield, Water and Nitrogen Use Efficiencies

Yuxi Li [1,2], Jian Chen [1,2], Longbing Tian [1,2], Zhaoyin Shen [1,2], Daniel Buchvaldt Amby [3,4], Fulai Liu [3], Qiang Gao [1,2] and Yin Wang [1,2,*]

1 College of Resources and Environmental Sciences, Jilin Agricultural University, Changchun 130118, China

2 Key Laboratory of Straw Comprehensive Utilization and Black Soil Conservation, Ministry of Education Jilin Agricultural University, Changchun 130118, China

3 Department of Plant and Environmental Science, Faculty of Science, University of Copenhagen, Højbakkegaard Alle 13, 2630 CopenhagenTaastrup, Denmark

4 Department of Plant and Environmental Sciences, Section for Organismal Biology, Faculty of Science, University of Copenhagen, Thorvaldsensvej 40, 1871 Frederiksberg, Denmark

* Correspondence: wangyin@jlau.edu.cn

Table S1 Summary of ANOVA for the effects of seedling-stage soil water level, N fertilization and experimental year on shoot and root dry biomass (DM) and their ratio at different growth stages, grain yield and yield components of maize plants

Source of variation	Shoot DM				Root DM			Root: shoot ratio			Grain yield	Ear number	Grain number per ear	100-grain weight
	BDI	ADI	R1	R6	BDI	ADI	R1	BDI	ADI	R1				
Water (W)	—	***	ns	ns	—	***	ns	—	ns	ns	ns	***	ns	ns
Nitrogen (N)	***	***	***	***	***	***	***	***	***	ns	***	***	***	***
Year (Y)	***	***	***	***	***	***	***	***	ns	**	***	***	***	***
W × N	—	ns	ns	ns	—	ns	ns	—	ns	ns	ns	*	ns	ns
W × Y	—	ns	**	**	—	**	**	—	*	ns	**	ns	**	**
N × Y	***	ns	ns	ns	***	ns	ns	ns	ns	ns	ns	*	ns	ns
W × N × Y	—	ns	ns	ns	—	ns	ns	—	ns	ns	ns	ns	ns	ns

DI, deficit irrigation; BDI, Before seedling-stage DI; ADI, After seedling-stage DI; R1, silking stage; R6, Maturity stage.

*, ** and *** indicate significance at $P<0.05$, $P<0.01$ and $P<0.001$, respectively, and ns indicates non-significance ($P > 0.05$).

Table S2 Summary of ANOVA for the effects of seedling-stage soil water level, N fertilization and experimental year on N uptake, N use efficiency (NUE), actual evapotranspiration (ETa) and water use efficiency (WUE) for shoot dry biomass (WUEdm) and grain yield (WUEgy) of maize plants at different growth stages

Source of variation	Plant N uptake				NUE				ETa					WUEdm / WUEgy				
	BDI	ADI	R1	R6	BDI	ADI	R1	R6	S-BDI	BDI-ADI	ADI-R1	R1-R6	S-R6	S-BDI	BDI-ADI	ADI-R1	R1-R6	S-R6
Water (W)	—	***	ns	ns	—	ns	ns	ns	—	***	*	ns	***	—	***	ns	ns	*
Nitrogen (N)	***	***	***	***	—	—	—	—	ns	ns	ns	ns	ns	***	***	***	***	***
Year (Y)	***	***	***	***	***	***	***	***	***	***	***	***	***	*	ns	***	***	*
W × N	—	ns	ns	ns	—	—	—	—	—	ns	ns	ns	ns	—	ns	ns	ns	ns
W × Y	—	ns	*	***	—	**	**	***	—	**	ns	ns	ns	—	**	ns	ns	*
N × Y	***	ns	*	**	—	—	—	—	ns	ns	ns	ns	ns	ns	ns	ns	ns	ns
W × N × Y	—	ns	*	*	—	—	—	—	—	ns	ns	ns	ns	—	ns	ns	ns	ns

S, sowing; DI, deficit irrigation; BDI, Before seedling-stage DI; ADI, After seedling-stage DI; R1, silking stage; R6, Maturity stage.

*, ** and *** indicate significance at P<0.05, P<0.01 and P<0.001, respectively, and ns indicates non-significance ($P > 0.05$).

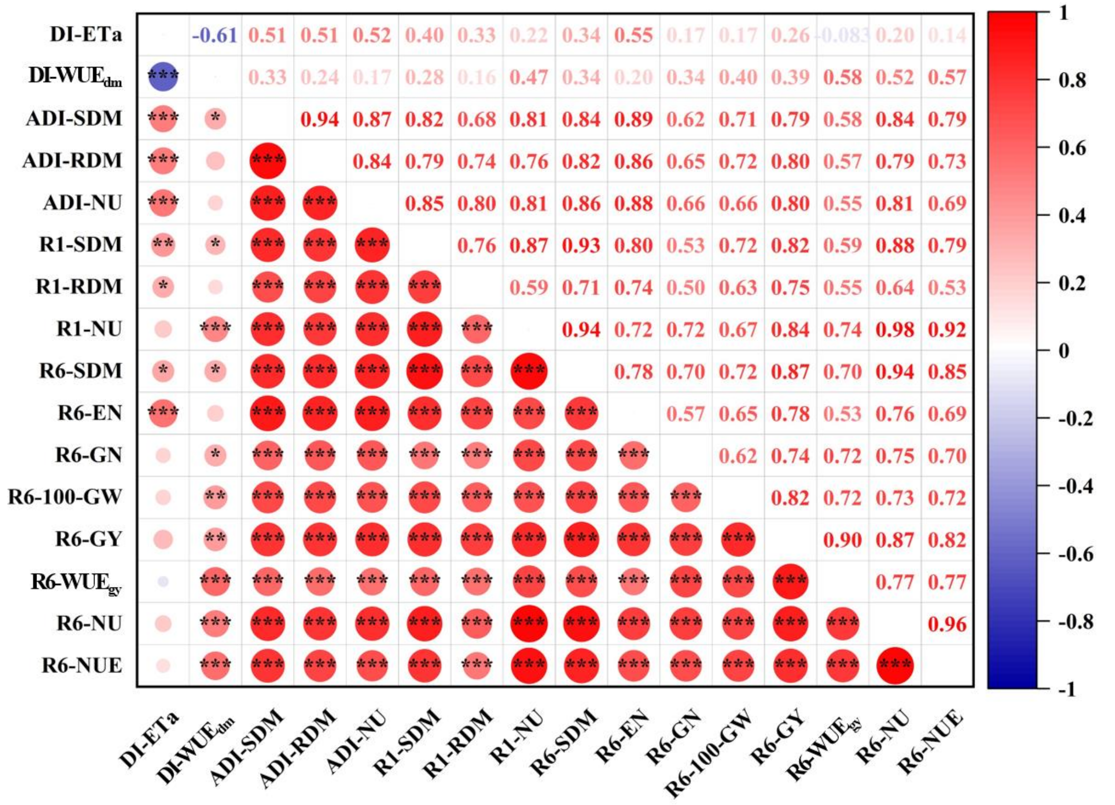

Figure S1 Correlations coefficients (upper triangular) and significant marks (lower triangular) among maize growth, water and N use parameters at different periods, respectively.

DI, deficit irrigation period; ADI, after seedling-stage DI; R1, silking stage; R6, maturity stage; ETa, actual evapotranspiration; SDM, shoot dry matter; RDM, root dry matter; NU, plant N uptake; NUE, N use efficiency; GY, grain yield; EN, ear number; GN, grain number per ear; 100-GW, 100-grain weight; WUEdm and WUEgy, water use efficiency for SDM and GY, respectively.

Significant mark *, ** and *** indicate significance level at *P*<0.05, *P*<0.01 and *P*<0.001, respectively.
